# Decoding Macrophage Dynamics: A Pathway to Understanding and Treating Inflammatory Skin Diseases

**DOI:** 10.3390/ijms26094287

**Published:** 2025-05-01

**Authors:** Shengliang Gu, Lei Xu, Bin Huang, Kai Xiong, Xuesong Yang, Jianzhou Ye

**Affiliations:** 1 The First School of Clinical Medicine, Yunnan University of Chinese Medicine, Kunming 650500, China; sliang1785@163.com (S.G.); xulei@ynucm.edu.cn (L.X.); huangbin@ynucm.edu.cn (B.H.); 2 Yunnan Provincial Clinical Medical Centre for Traditional Chinese Medicine Project (Dermatology), Kunming 650500, China; 3 The First School of Clinical Medicine, Guizhou University of Chinese Medicine, Guiyang 550025, China; a1255354266@163.com

**Keywords:** inflammatory dermatosis, macrophage polarization, epigenetics, atopic dermatitis, psoriasis

## Abstract

Psoriasis and atopic dermatitis (AD) are both chronic inflammatory skin diseases. Their pathogenesis remains incompletely understood. The polarization states of macrophages, as a crucial part of the innate immune system, are influenced by various factors such as cytokines, inflammatory mediators, and epigenetics. Research has demonstrated that macrophages play a “double-edged sword” role in the pathological process of inflammatory skin diseases: they both drive inflammation progression and participate in tissue repair. This article summarizes the roles of macrophages in the inflammatory development and tissue homeostasis of psoriasis and atopic dermatitis. It explores the impact of different factors on macrophages and inflammatory skin diseases. In conclusion, understanding the classification and plasticity of macrophages is crucial for a deeper understanding of the pathogenesis of psoriasis and AD and the development of personalized treatments.

## 1. Introduction

Inflammation is a basic physiological and pathological process, as well as a conserved defense mechanism. Persistent tissue stress and immune system dysfunction can lead to more complex pathological changes, which are associated with chronic inflammation [1]. Atopic dermatitis and psoriasis are chronic inflammatory skin diseases linked to autoimmunity, and their prevalence is increasing [2,3]. The difference is that AD is characterized by an over-activated Th2 immune response that drives inflammatory signals such as IL-4 and IL-13, while in psoriasis, the Th17 immune response and related IL-17 cytokine activation are at the core of pathogenesis research [4]. Both diseases require long-term, ongoing treatment to manage the condition, reduce the risk of recurrence, and address any existing or potential complications. For patients with more severe disease, immunosuppressive or immunomodulatory treatment options should be considered. However, while targeted drugs represent advances in treatment, a significant number of patients still fail to benefit from a given therapy.

Macrophages are immune cells that play a key role in inflammation and tissue homeostasis, accumulating in acute and chronically inflamed skin [5]. The strong plasticity of macrophages enables them to play different roles at various stages of inflammation [6]. Different cytokines polarize macrophages into two major classes: M1 and M2 phenotypes. The M1 type promotes the development of inflammatory responses by enhancing the spread of inflammation and increasing the level of antigen presentation. In contrast, M2 has anti-inflammatory and immunomodulatory effects and plays an important role in type 2 inflammation [7,8,9]. In chronic inflammatory skin diseases, macrophage activation is increased, leading to the continued release of growth factors and chemokines that transmit inflammatory signals and further activate the immune response [10]. Factors such as an overactive inflammatory response, abnormal cytokine production, and impaired anti-inflammatory regulation may cause macrophage dysfunction and an imbalances in the immune response, contributing to the development of the disease [9].

With multiple explorations of macrophages in the treatment and diagnosis of various inflammation-related diseases, new approaches have provided a deeper understanding of the developmental trajectories, transcriptional programs, and life cycles of these cells [11,12,13,14,15,16]. There is growing evidence that differences in the origin, phenotype, and function of macrophages play an important role in both physiological and pathogenic inflammation. In this review, we discuss recent progress regarding macrophages in the development of chronic inflammatory skin diseases, the relevant signals associated with macrophages in these conditions, and their potential as therapeutic targets.

## 2. Macrophages Overview

### 2.1. Function and Characteristics of Macrophages

There are two main sources of macrophages in the body: embryonically derived tissue-resident macrophages and bone marrow-derived mononuclear macrophages [17]. During aging or pathological inflammation, the balance of tissue-resident macrophages is disrupted, and a certain proportion of circulating mononuclear macrophages are recruited into specific tissues by disease-related signals, where they differentiate and supplement the population of tissue-resident macrophages [18]. Macrophages exhibit functional and structural differences in different tissues and organs, which could be associated with their source and environmental conditions [19,20,21]. Kupffer cells are liver macrophages primarily responsible for removing bacteria, viruses, waste products, and aging red blood cells from the blood. They are also involved in regulating liver immune responses and inflammatory responses [22]. Microglia, the macrophages in the central nervous system, are essential for nerve development, repair, and the preservation of nerve tissue homeostasis through their interactions with neurons [23,24]. Alveolar macrophages are capable of phagocytosis and the removal of inhaled pathogens, dust, and cell debris. They participate in the lung immune response to prevent the respiratory system from infections and damage [25]. In the process of angiogenesis, macrophages can produce angiogenic factors, promote the formation of new blood vessels, and participate in the stability and remodeling of blood vessels [26]. This indicates that macrophages are diverse and plastic, have tissue-specific responses, and also retain their core phagocytic function [26].

### 2.2. Polarization of Macrophages

The function of macrophages in vivo shows dynamic changes and can be polarized divided into different phenotypes when stimulated by the surrounding microenvironment [6]. Nowadays, scientists have reached a basic consensus on the classification of macrophage activation phenotypes, and understanding the classical types of macrophage activation remains key to comprehending macrophage function. In the polarization response, classically activated M1 macrophages are stimulated by TNF-α, LPS, Pathogen-Associated Molecular Patterns (PAMPs), and IFN-γ. They also release high levels of pro-inflammatory factors such as interleukin-6 (IL-6) [27,28,29]. Studies have shown that M1 macrophages upregulate the expression of suppressors of cytokine signaling 3 (SOCS3), activate inducible nitric oxide synthase (iNOS), utilize L-arginine as a substrate to produce nitric oxide (NO), and enhance antimicrobial activity and the pro-inflammatory response [30,31,32]. M1 macrophages have bactericidal and anti-tumor activity, can promote inflammation amplification, have a high antigen presentation capacity, and activate Th1 immunity in response to infection [33,34].

Replacement-activated M2 macrophages are induced by IL-4, IL-10, and IL-13 signals [35]. They express high levels of Mannose Receptor CD206, IL-1 receptor antagonist (IL1Ra), and Arginase 1 (Arg1), possess a greater immunosuppressive power, and activate Th2 immunity [36]. Arg1 breaks down L-arginine into L-ornithine, a precursor of polyamines and L-proline. Polyamines play an important role in cell proliferation, while L-proline is an essential amino acid that contributes to collagen synthesis in tissue repair [37]. Therefore, M2 macrophages play a role in the regression of inflammation as well as in tissue healing and repair.

Polarization-related cytokines may coexist with various pathogens or microbiome components in the inflammatory environment, leading to a more nuanced phenotype and function of macrophages involved in disease progression. M2-type macrophages can be further divided into four subgroups: M2a, M2b, M2c, and M2d. These subgroups are classified based on their distinct functions and characteristics in various inflammatory and tissue repair settings [38]. M2a macrophages are mainly induced by IL-4 and IL-13 and express high levels of CD206. They can promote angiogenesis, fibroblast activation, and collagen production, which are conducive to tissue repair and reconstruction [39]. M2a macrophages play an important role in chronic wound healing, tissue regeneration, and fibrosis [40]. Through the activation of immune complexes and TLR agonists, M2b macrophages suppress the immune response, reduce inflammation, and help maintain immune system balance by secreting anti-inflammatory molecules such as IL-10 and TGF-β [41,42]. This subgroup plays an important role in the regulation of autoimmune diseases and allergic reactions. M2c macrophages secrete anti-inflammatory and pro-phagocytic cytokines that play a key role in the prevention and treatment of certain infectious diseases. They are characterized by high levels of expression of the scavenger receptor CD163 [43,44,45]. M2d macrophages are also involved in immune regulation and have been implicated in tissue repair, angiogenesis, and tumor progression [46,47].

Macrophage infiltration and polarization are critical to the progression and balance of inflammation and are influenced by factors such as the levels of cytokines and microbial products released during inflammation, and the duration of exposure. At the same time, epigenetic modifications and autophagy regulation also affect the polarization of macrophages and their ability to respond to infection.

## 3. The Role of Macrophages in Skin Injury

Skin is the body’s first line of defense against external pathogens and microorganisms. Its integrity and normal immune response are critical to host health. Immune dysregulation leads to chronic inflammatory and autoimmune diseases such as atopic dermatitis, psoriasis, contact dermatitis, and rosacea [48,49,50,51]. Previous studies of these diseases have focused on the function of Langerhans cells, keratinocytes, and T cells, but there are other types of immune cells in the skin that work together to maintain skin immune homeostasis [52,53]. For example, in a model of aseptic inflammation, epithelial cells activate tissue-resident and monocyte-derived macrophages through cyclooxygenase 2 expression. Activated macrophages become polarized to the M2 phenotype in response to epithelial inflammation, and this epithelial–macrophage crosstalk leads to extracellular matrix (ECM) remodeling and aggravated basement membrane destruction [54,55]. Keratinocytes promote macrophage polarization through the TGFβR1 signaling pathway, release LRG1-rich extracellular vesicles, and promote skin inflammation [56]. Treg lymphocytes reduce the severity of psoriasis by inhibiting the number and pro-inflammatory activity of macrophages [57]. High IL-1 signaling drives abnormal Arg1^Hi^ macrophage–fibroblast communication, leading to delayed wound healing in aging skin [58]. These studies suggest that the complex communication between macrophages and other cells plays a role in skin diseases.

Macrophages also play an important role in tissue repair and wound healing, and expressing different phenotypes according to different stages of wound healing is the key to ensuring normal healing. When the skin is injured, circulating monocytes are activated by the wound microenvironment to become M1 macrophages [59]. These macrophages clear and phagocytose dead cells, bacteria, and foreign bodies, and they also present antigens to activate other immune cells. During the inflammatory phase, inflammatory factors (such as TNF-α, IL-1β, IL-6, etc.) are released to initiate the inflammatory response. In the repair phase, following the conclusion of the inflammatory phase, M1-type macrophages transform into M2-type macrophages, releasing various growth factors, chemokines, and metabolites to stimulate the proliferation of fibroblasts and keratinocytes, as well as to promote collagen synthesis and granulation tissue formation [60,61]. During the remodeling phase, macrophages promote angiogenesis, providing the large amounts of oxygen and nutrients needed for cell proliferation and tissue reconstruction [62,63]. An insufficient or dysfunctional number of macrophages can affect wound healing, increasing the risk of infection and further leading to other diseases.

When there are pathological factors such as chronic inflammation, diabetes, and malnutrition, the function of macrophages is affected, which in turn impacts tissue repair and leads to poor wound healing. For example, a long-term hyperglycemic microenvironment and abnormal metabolic conditions can result in the abnormal polarization of macrophages, irregular secretion of cytokines, and impaired phagocytosis and clearance functions. This can cause sustained oxidative stress and inflammatory responses at the wound site, significantly prolonging the inflammatory phase and delaying or disrupting the proliferative and reconstruction phases, ultimately leading to delayed or non-healing of the wound [59,64,65]. Additionally, the increased inflammatory response may further damage the skin barrier function and exacerbate the disease process. Therefore, an in-depth study of the physiological functions and plasticity of macrophages is of great significance for wound healing and the treatment of chronic inflammatory skin diseases.

## 4. The Role of Macrophages in AD and Psoriasis

Chronic inflammatory skin diseases not only affect the physical health of patients but can also lead to psychological burdens and social dysfunction. As populations age and environmental factors change, the incidence of common inflammatory skin diseases such as psoriasis and AD is on the rise, putting tremendous pressure on healthcare systems [3,66]. In recent years, with advances in immunology, molecular biology, and genetics, we have gained a deeper understanding of the involvement of macrophages in the pathophysiology and clinical manifestations of psoriasis and atopic dermatitis.

**Figure 1:**
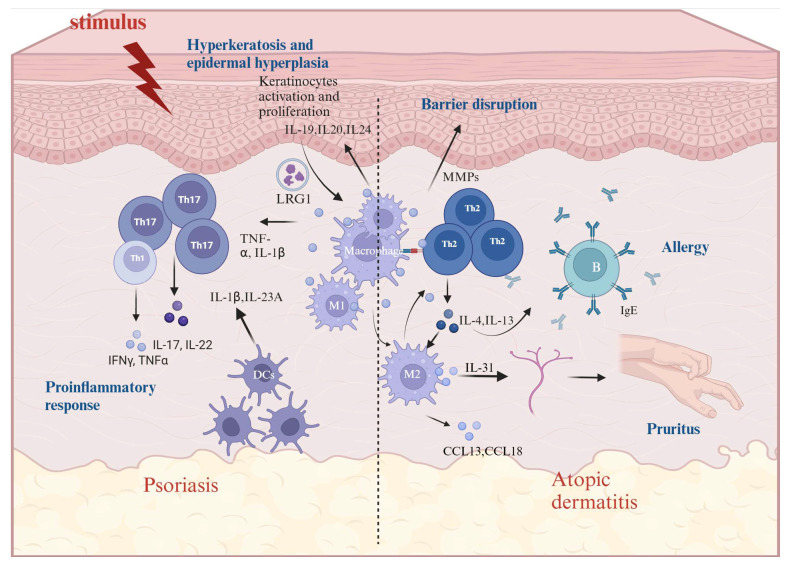
Macrophages are involved in the progression of two inflammatory skin diseases, AD and psoriasis. They are activated into various phenotypes, participating in the progression of both diseases through the secretion of multiple cytokines and chemokines, as well as interactions with other cells.

According to previous clinical data analysis, macrophage markers and inflammatory cytokines such as TNF-α, IL-1β, IL-17A, and IL-23A are highly expressed in skin samples from psoriasis patients [67]. In addition, compared with the normal control group, the ratio of M1 to M2 macrophages in the tissue of mice in a psoriasis model was higher, and macrophage depletion could effectively reduce skin thickness and improve psoriasis symptoms [68]. This evidence suggests that macrophages are involved in the pathogenesis and maintenance of psoriasis, and that M1-type polarization may be a key factor in this process. Activated macrophages release cytokines through the activation of NF-κB, promoting the expression of inflammatory cytokines, chemokines, and antimicrobial peptides, which leads to the aggravation of the inflammatory response in psoriasis [69]. At the same time, macrophages promote the proliferation and differentiation of keratinocytes, leading to the characteristic pathological changes associated with psoriasis, namely hyperkeratosis, epidermal hyperplasia, and vasodilation. In addition to releasing numerous inflammatory mediators, macrophages release numerous proteases, such as matrix metalloproteinases (MMPs), which can degrade collagen in the skin’s basement membrane, disrupt the skin barrier, exacerbate the inflammatory response, and promote disease development (Figure 1). The NLRP3 inflammasome in macrophages is activated directly or indirectly by a variety of stimuli, such as pathogen-associated molecular patterns (PAMPs), damage-associated molecular patterns (DAMPs), and cytokines like IL-1β and TNF-α. These activation signals cause the NLRP3 protein to assemble with the ASC protein to form the inflammasome, induce Caspase-1 self-cleavage and activation, and further mediate the maturation of the pro-inflammatory factors IL-1β and IL-18 [70,71].

Macrophages are involved in nearly the entire process of the pathogenesis of AD, and their activation has been observed in both the acute and chronic phases. AD patients exhibit excessive Th2 type immunity [72]. Th2 lymphocytes release high levels of IL-4, IL-5, IL-13, and IL-31, promoting the polarization of M2 macrophages. M2 macrophages interact with Th2 cells and fibroblasts via ligand–receptor signaling to spread type 2 inflammation in AD [73]. The stimulation of the allergen causes dysregulation of the innate immune system, resulting in damage to the skin barrier, which may be the first step in the onset of AD [74]. Toll-like receptors (TLRs), which recognize the signature components of bacteria, viruses, and other pathogens, play a key role in the innate immune system. TLRs mainly stimulate the activation of the transcription factor NF-κB through MyD88 signaling. The translocation of NF-κB into the nucleus promotes the expression and release of pro-inflammatory cytokines such as TNF-α, IL-6, and IL-1β [75]. The lowered expression of TLR2 in the macrophages of AD patients causes a decrease in M1-type polarization, limits the pro-inflammatory response in conjunction with CD14, and raises the risk of Staphylococcus aureus infection, which might encourage a Th2 response [76,77]. Here, there is a complex mutual feedback loop between skin barrier damage and inflammation, which together promote the occurrence of chronic inflammation and sustained damage. When the skin barrier is damaged, cell tight junctions and the breakdown of desmosomes allow foreign antigens to penetrate the skin. Skin-resident macrophages sense and phagocytose these foreign antigens before migrating to the lymph nodes [55]. Macrophages process and load foreign antigens onto major histocompatibility complex (MHC) Class II molecules. The antigens carried by MHC Class II molecules are then presented to T helper cells, primarily TH2 cells. Upon activation, TH2 cells secrete cytokines that stimulate B cells to produce IgE. The binding of IgE to the high-affinity IgE receptor (FcεRI) on mast cells and basophils results in the release of inflammatory mediators that trigger acute AD symptoms [78] (Figure 1).

## 5. The Role of Macrophages in Inflammatory Pruritus

Pruritus is a typical clinical symptom of chronic inflammatory skin diseases. In states of pathological skin inflammation, pain and pruritus can be abnormally exacerbated. Patients may have a lower threshold for pruritus, which can be triggered by mild stimulation. This itching prompts scratching, which excites nerve fibers and creates a vicious itch–scratch cycle [79]. This vicious cycle leads to severe skin barrier breakdown, further aggravating itching and inflammation. It is important to note that the mechanisms that lead to itching and the itch–scratch cycle are multifactorial, influenced by interactions between the skin microbiome, the epidermal barrier, immune cells, and sensory nerves [80]. Sensory nerves are in close contact with infiltrating immune cells and epithelial cells, and they interact with a variety of neurotransmitters to carry nerve impulses from the peripheral nervous system along C fibers to the spinal cord and eventually to the somatosensory cortex of the brain [81]. The neurosensory signal transmission of itch depends on G-protein-coupled receptors and potential transient receptor channels. Type 2 cytokines such as IL-4, IL-13, IL-31, and thymic stromal lymphopoietin are important pruritus mediators [82,83].

IL-31 causes itching by stimulating peripheral sensory nerve fibers in the skin. It was previously thought that activated TH2 cells were the primary cellular source of IL-31; however, recent studies have shown that mast cells, basophils, and macrophages also secrete IL-31 [84]. The expression of IL-31 in M2 macrophages is closely related to keratinocyte-derived TSLP and dermal fibroblast-derived periostin. Blocking IL-31 signaling or directly depleting macrophages can alleviate pruritus. At the same time, basophils promote the secretion of IL-31 from macrophages, which plays a role in the pruritus response. This evidence suggests that macrophages are involved in the pruritus response through signal crosstalk with other immune cells and epithelial cells [85]. TRPV4 is an ion channel that belongs to the transient receptor potential (TRP) channel family. TRPV4 is sensitive to changes in osmotic pressure, temperature, and both chemical and mechanical stimulation. The activation of TRPV4 leads to an increase in intracellular calcium ion concentration, which then activates a series of downstream signaling pathways that ultimately result in changes to cell function. Activation of TRPV4 in the skin can lead to inflammation, itching, vasodilation, and other responses [86,87,88]. TRPV4 is selectively expressed in dermal macrophages and keratinocytes and may act as a pro-inflammatory molecule in macrophages, participating in various pathological processes associated with inflammatory diseases [89,90]. Studies have shown that TRPV4 in macrophages causes spontaneous scratching associated with SADBE-induced atopic dermatitis. A loss of TRPV4 in macrophages suppresses pruritus. In skin biopsy samples from patients with chronic idiopathic pruritus, the expression level of TRPV4 was significantly higher than in samples from healthy controls. In addition, their research suggests that the serotonin signaling pathway is a key downstream mechanism for both allergic and non-allergic pruritus mediated by TRPV4 [89,91]. Other studies showed that intervention with the 5-HT2A receptor or TRPV4 inhibitor significantly reduced scratching behavior in mice, suggesting that 5-HT2A and TRPV4 may be new targets for inhibiting itch symptoms in AD [92]. Macrophage autophagy increases the release of IL-10 in the skin tissue and enhances the expression of key genes in the signaling of sensory neurons and sensitizing these nerves. Stimulating macrophage autophagy can promote wound healing and alleviate pruritus [93].

The pruritus mechanisms in chronic inflammatory skin diseases involve complex immune responses and nerve signaling. During this process, macrophages significantly affect the occurrence and persistence of pruritus by regulating the local environment, releasing cytokines, and altering tissue structure. Therefore, targeting macrophages and the mediators they secrete may help alleviate pruritus symptoms in chronic inflammatory skin diseases.

## 6. Factors Influencing Macrophage Polarization in AD and Psoriasis

In the special pathological process within the skin, macrophages are recruited to the diseased site and polarized into various phenotypes with different functions under the influence of the microenvironment. Macrophages with different phenotypes release various signals to promote or inhibit the repair and proliferation of surrounding tissues. To date, many studies on chronic inflammatory skin diseases have focused on phenotypic changes in macrophages and the unbalanced M1/M2 polarization ratio [94,95,96,97]. Therefore, regulating M1 and M2 macrophage polarization and their ratio is expected to be a new therapeutic approach for chronic inflammatory skin diseases. In the following, we discuss some of the factors that influence macrophage polarization in specific dermatitis and psoriasis.

### 6.1. Cell-to-Cell Cross-Talk

In psoriasis, T cells induce the differentiation of macrophages into the M1 type by secreting IFN-γ, enhancing their phagocytic and inflammatory cytokine release functions. At the same time, M1 secretion of TNF-α and IL-18 and other pro-inflammatory factors promote Th1/Th17 cells to secrete IL-17A, IFN-γ and IL-21 and other inflammatory factors, which aggravates the inflammatory response of psoriasis [98]. Several recent studies have shown that there is a signaling crosstalk between keratinocytes and macrophages that promotes skin inflammation. Keratinocyte-derived leucine-rich alpha-2-glycoprotein 1 (Lrg1) extracellular vesicles induce the expression and release of pro-inflammatory cytokines such as IL-1β and TNF-α in macrophages. In addition, the extracellular vesicles can induce M0 macrophages to polarize into the M1 type, which is dependent on the TGFβ receptor 1 (TGFβR1) pathway [56]. This is a novel mechanism for the pathogenesis of psoriatic dermatitis and a potential emerging therapeutic target. High Mobility Group Box 1 (HMGB1) is a pro-inflammatory factor produced by keratinocytes and is highly expressed in psoriasis model mice. It not only promotes the excessive proliferation of keratinocytes and the expression of pro-inflammatory factors, but also induces the polarization of M1-type macrophages and increases the ratio of M1 to M2, leading to the exacerbation of psoriasis [99]. Treg-of-B cells inhibit NLRP3 inflammasome activation by inhibiting NF-κB signaling, inducing STAT6 phosphorylation in macrophages, and promoting M2 macrophage polarization, thereby improving psoriasis symptoms and reducing inflammation [100].

In AD inflammation, macrophages become M2 activated by IL-4 or IL-13 produced by Th2 cells, mast cells, and basophils [101]. The expressions of MMP12 and CCL18 are increased in patients with AD. Histamine and inflammatory factors secreted by TH2 cells up-regulate the expression of MMP12 and CCL18 in M2 macrophages, thereby promoting the progression of AD [102,103]. It has been shown that TNF-α released by activated macrophages binds to receptors in keratinocytes and induces keratinocyte proliferation by enhancing chemokine production. Blocking the interaction between macrophages and keratinocytes can reduce inflammation in the co-culture system of macrophages and keratinocytes [104]. In addition, one study found that S1 macrophages, characterized by FRβ/CD163 expression, play a role in maintaining tissue homeostasis and ensuring the renewal of eosinophil monocytes and eosinophils. In AD, the S1 phenotype is suppressed, and its development is regulated by keratinocytes and fibroblast laminin. Treatment with exogenous laminin-α can induce monocytes to become S1 type, thereby reducing inflammation and alleviating AD pathology [105].

### 6.2. Epigenetic Regulation of Macrophages

Epigenetic alterations have been identified as important underlying factors in the pathogenesis of many chronic inflammatory skin diseases [106]. Epigenetic modifications, such as DNA methylation, histone modifications, and non-coding RNAs, are involved in skin barrier function and immune response in psoriasis and atopic dermatitis [107]. In chronic inflammation, epigenetic modifications are involved in the differentiation and activation of immune cells, facilitating the dynamic regulation of the polarization gene network and signaling cascades of macrophages. These modifications are considered key determinants of the functional regulation and heterogeneity of macrophages [108]. As a key regulator of macrophage polarization, epigenetic regulatory mechanisms affect the occurrence and resolution of skin inflammation by dynamically regulating the balance of macrophage subsets [109,110]. Here, we investigate multiple well-examined epigenetic modifications that have been shown to significantly influence macrophage polarization and disease progression in psoriasis.

#### 6.2.1. DNA Methylation

DNA methylation is an important epigenetic modification that regulates gene expression by adding methyl groups to specific locations of DNA molecules [111,112]. In mammals, DNA methylation primarily occurs on the fifth carbon atom of cytosine (C), forming 5-methylcytosine (5mC) [113]. This modification typically takes place in CpG dinucleotide sequences, where the methylation status of the CpG island is closely related to the transcriptional activity of the associated gene [114]. When the CpG island is hypermethylated, it often leads to chromatin condensation, hindering the binding of transcription factors to DNA and thereby inhibiting gene transcription, which is associated with transcriptional silencing [115]. DNMT (DNA methyltransferase) is a key enzyme that catalyzes DNA methylation. It is capable of adding methyl groups to the 5’ position of cytosine in CpG dinucleotides, thereby facilitating the methylation modification of DNA. Three types of DNMT with catalytic activity exist in mammals, namely DNMT1, DNMT3A, and DNMT3B [116,117].

Abnormal DNA methylation is closely related to the occurrence and development of many diseases. In the AD-like mouse model, DNMT1 was reduced, leading to hypomethylation of the C-C chemokine receptor type 7 (CCR7). The expression of CCR7 in skin dendritic cells (DCs) is up-regulated by hypomethylation. This suggests that changes in DNMT1 levels may be closely related to the methylation status of specific receptors and the subsequent immune response [118]. Interestingly, recent studies have shown an association between DNA methylation and the differential expression of M1 and M2 macrophages. By decreasing the expression of DNMT3a/b, the DNA methylation of the GPX1 promoter is reduced, thereby inhibiting AGEs-induced M1 polarization of macrophages [119]. In a study of obesity-induced inflammation, it was found that the use of drugs to inhibit DNA methylation, or the genetic deletion of DNMT1, promoted the alternative activation of macrophages and suppressed inflammation [120]. These findings suggest that DNA methyltransferase plays a role in the regulation of macrophage polarization, which may be a new strategy to prevent and treat diseases related to macrophage polarization imbalance.

#### 6.2.2. miRNA

miRNAs are a class of non-coding RNAs with a length of about 22 nucleotides. They are mainly transcribed into primary miRNA (pri-miRNA) by RNA polymerase II in the nucleus and then processed into precursor miRNA (pre-miRNA) under the action of the Drosha enzyme. After being transported to the cytoplasm, the pre-miRNA is further processed to form mature miRNA under the action of the Dicer enzyme [121,122,123]. Mature miRNAs bind to Argonaute (AGO) proteins to form the RNA-induced silencing complex (RISC), exerting their regulatory effects on target genes and cellular functions [124]. Recent studies have shown that miRNAs have become active in regulating the expression of key genes that affect the polarization of macrophages and the response to inflammation [125,126]. They also participate in the immune response by regulating different transcription factors [127,128].

miR-146a is involved in the proliferation of keratinocytes and fibroblasts, as well as the production of inflammatory factors [129]. It plays a role in innate immunity and influences inflammation by regulating macrophage polarization [130]. When the expression of miR-146a is up-regulated, it inhibits the expression of Notch1, which subsequently affects the downstream signaling pathway, thereby playing a negative regulatory role in the polarization of M1-type macrophages. Additionally, the inhibition of PPARγ by miR-146a may disrupt the normal polarization of M2-type macrophages [131,132]. The study demonstrated that the expression of miR-146a may be related to the early onset and severity of psoriasis [133]. In an miR-146a deficient AD mouse model, a stronger and earlier inflammatory response was observed [134]. miR-146a targets key molecules in the TLR and IL-1R signaling pathways, such as IL-1 receptor-associated kinase 1 (IRAK1) and TNF receptor-associated factor 6 (TRAF6) [135]. These molecules play a crucial role in the initiation and maintenance of inflammation in psoriasis. Additionally, alterations in the expression of miR-146a affect the secretion of pro-inflammatory cytokines, including IL-6, IL-8, CCL5, and ubiquitin D. miR-146a controls the intensity of the inflammatory response and disease progression in AD and psoriasis through the negative feedback regulation of inflammatory signaling pathways and its influence on macrophage polarization [136].

miR-155 is an important immunomodulator in macrophages. Under the stimulation of LPS and IFN-γ, the expression of miR-155 is up-regulated to regulate the function of macrophages by targeting SHIP1. SHIP1 is a negative regulator of the PI3K-Akt signaling pathway. The inhibition of SHIP1 by miR-155 activates the PI3K-Akt signaling pathway, thus promoting the M1-type polarization of macrophages. M1 macrophages can secrete a large number of pro-inflammatory cytokines, such as TNF-α and IL-1β, which enhance the body’s resistance to pathogens [137,138,139,140]. miR-155 promotes the expression of BCL6, which is a negative transcription factor in the NF-κB pathway. During the polarization of M1 macrophages, BCL6 can finely regulate the NF-κB pathway and prevent overactivation of the inflammatory response [141]. In addition, miR-155 is also necessary for Th17 and Treg cell differentiation, and increased expression of miR-155 inhibits CTLA-4 activity in atopic dermatitis, leading to abnormal T cell proliferation and persistent inflammation, which are positively correlated with disease severity [142]. miR-155 is involved in the proliferation and differentiation of keratinocytes, as well as the regulation of the immune response [143]. Studies have shown that miR-155 is highly expressed in psoriasis and is correlated with the severity of the disease. It not only regulates the genes PTEN, AKT, Bax, and Bcl-2, but also promotes the differentiation of Th17 cells and the expression of related cytokines. Additionally, it regulates the Th1/Th2 ratio, thereby increasing the secretion of pro-inflammatory cytokines and aggravating skin inflammation [144,145]. In addition, miRNA-155 has been observed to be associated with keratinocyte differentiation in psoriasis [146].

miR-21 is highly expressed in macrophages and dendritic cells. In the skin of psoriasis patients, the up-regulated expression of miR-21 inhibits T cell apoptosis and promotes the excessive proliferation of keratinocytes, which may lead to psoriatic skin inflammation [147]. In addition, IL-22 induces miR-21-3p through the STAT3/NF-κB pathway, and the overexpression of miR-21-3p results in global changes in keratinocyte transcriptome characteristics [148]. Although research into the correlation between miR-21 and AD has not been in-depth, the expression of miR-21 is up-regulated in a variety of Th2-related diseases. It has been found that miR-21 may regulate the balance of Th1/Th2 by targeting IL-12p35 [149,150]. Macrophage CSF-1R pTyr-721 signaling inhibits inflammatory phenotypes mainly by inducing miR-21, which positively regulates the expression of M2 markers. An analysis of CSF-1R-regulated miR-21-targeted messenger RNAs found that 80% of the classical miR-21 targets regulated by CSF-1 were pro-inflammatory molecules. This suggests that miR-21 promotes the polarization of M2 macrophages by inhibiting the expression of pro-inflammatory molecules [151].

### 6.3. Other Factors

TLRs are the core molecules of macrophages that recognize pathogens, regulate immune responses, and play an important role in psoriasis. Studies have shown that TLR7-9 ligands activate M1 macrophage polarization, and TLR7 agonists lead to a higher M1/M2 macrophage ratio in psoriatic lesions. By depleting macrophages in mice and blocking M1 macrophage polarization, psoriatic-like inflammation induced by TLR7-9 can be improved [68]. Calcium/calmodulin-dependent protein kinase IV (CaMK4) plays a key role in autoimmune diseases, significantly affecting genes involved in immune response and inflammation. Studies have shown that CaMK4 is highly expressed in psoriatic skin lesions. It stimulates T cells to release IL-17A through the CaMK4/AKT/NF-κB pathway, which promotes the excessive proliferation of keratinocytes, produces many antimicrobial peptides and chemokines, and recruits immune cells. In addition, CaMK4 can inhibit the production of IL-10 by macrophages through the ADCY1-cAMP-Erk1/2 and p38 pathways. This inhibition limits the polarization and phagocytosis of M2 macrophages, thereby promoting the development of psoriasis [152].

Single-cell transcriptome sequencing from AD patients showed that the expression of the glucose transporter GLUT3 was increased in M2 macrophages, which is necessary for their polarization and maintenance functions. It was found that GLUT3 directly interacts with RAS through its intracytoplasmic ring domain to regulate endocytosis and IL-4/STAT6 signaling activation, which is the main signaling pathway used for M2 polarization. In a mouse model of AD with conditional Glut3 deficiency, there was a notable decrease in M2-type macrophages, and inflammation, skin thickening, and keratosis were greatly reduced, resulting in alleviated AD symptoms [153]. Similar to the results of this study, a study on the role of macrophage autophagy in AD development found that knocking out the autophagy gene in macrophages alleviated AD symptoms in mice, and reduced M2 macrophage infiltration was observed in the lesions. Further studies showed that autophagy defects up-regulated the mRNA levels of SOCS1 and SOCS3, resulting in the inhibition of IL-4-Stat6-mediated M2 activation. These results suggest that defects in macrophage autophagy lead to decreased M2 activation and infiltration, indicating that the regulation of macrophage autophagy may be a promising target for the treatment of AD [154]. However, studies have identified a circRNA (hsa_circ_0004287) that is mainly expressed by macrophages in inflammatory states and is up-regulated in the peripheral blood mononuclear cells of both AD and psoriasis patients. hsa_circ_0004287 inhibited M1 macrophage activation and reduced skin inflammation in mice with AD and psoriasis [155]. This seems to be in contrast with the findings from the two studies referenced earlier, possibly due to the dynamic nature of macrophage polarization in inflammatory conditions, where the two macrophage types play different roles at different stages of AD inflammation. In AD, the macrophage phenotype evolves over time and is linked to the tissue microenvironment. Precisely evaluating this dynamic shift is challenging, and numerous experiments focus on polarization at a single stage of the process. Consequently, focusing on regulating the balance between the two types of macrophages may be more significant than just inhibiting or promoting one type.

## 7. Conclusions and Future Perspectives

Macrophages exhibit strong plasticity and can differentiate into various phenotypes, such as M1 and M2. In the past few decades, significant progress has been made in studying the origin, characteristics, and functions of macrophages. Recent multi-omics reference maps of prenatal skin have identified macrophages and the growth factors they produce as key drivers of endothelial development and blood vessel formation during human skin development [156]. Macrophages not only exert their immune functions to protect the skin from infection but also play an important role in skin morphogenesis, including hair follicle formation, scar-free skin healing, and angiogenesis, by interacting with non-immune cells [5,58,157]. With the rapid development of emerging technologies such as single-cell sequencing and spatial transcriptomics, the heterogeneity of macrophages, cell–cell interactions, and their spatial and temporal distribution in the skin microenvironment have been accurately analyzed, contributing to our understanding of the pathogenesis of chronic inflammatory skin diseases. For example, a recent study on psoriasis reported that keratinocytes promote the polarization of macrophages by secreting extracellular vesicles (EVs), thereby contributing to the development of psoriatic-like dermatitis [99]. This is a novel cellular communication mechanism that may become a potential therapeutic target for the treatment of psoriasis [158]. In the complex tissue environment, macrophages exhibit a variety of activation properties. In addition to the common cytokines present in the microenvironment, many studies have shown that factors such as intercellular crosstalk and epigenetic modifications influence the activation state of macrophages. Recent studies have also found that apoptotic cell types and selective phagocytosis receptors determine the subsequent transcriptional and functional responses of macrophages, thus affecting the outcome of immune responses in disease [159].

Due to the ongoing interactions between various cells during disease pathology, certain drugs may target multiple pathogenic links to exert their effects. Keratinocyte proliferation and macrophage activation can lead to epidermal hyperplasia in psoriasis-like skin. *Scutellaria baicalensis* Georgi can regulate oxidative stress and inflammatory signals, inhibit the inflammatory response associated with macrophage activation, and subsequently prevent the proliferation of keratinocytes, thereby alleviating the progression of psoriasis [160]. *Forsythia velutina* Nakai extract was able to reduce not only the infiltration of macrophages in AD but also the release of pro-inflammatory factors such as IL-1β and TNF-α, which act on keratinocytes to affect epidermal tight junctions [161]. This suggests that *Forsythia velutina* Nakai extract not only acts on individual cell types but also affects the crosstalk between AD-related cells. In addition, in the treatment of atopic dermatitis, anti-IL-4Rα can significantly reduce the expression of type II inflammation and affect the expression of T cells and dendritic cell-related genes [162]. However, other studies have shown that IL-4Rα treatment can lead to an increase in blood eosinophils [163,164]. Therefore, we believe that in future research, it is equally important to further explore the interaction of various systems in the pathogenesis of the disease and emphasize that targeted interventions need to balance the two-way influence of “effect–compensation”. Based on this, finding multi-target interventions may have more profound clinical significance.

## Abbreviations

The following abbreviations are used in this manuscript:

AD	Atopic dermatitis
LPS	Lipopolysaccharide
IFN-γ	Interferon-gamma
iNOS	Inducible nitric oxide synthase
PAMPs	Pathogen-Associated Molecular Patterns
SOCS3	Suppressors Of Cytokine Signaling 3
ECM	Extracellular matrix
DNMT	DNA methyltransferase
MHC	Major histocompatibility complex
Arg1	Arginase 1
MMPs	Matrix metalloproteinases
CaMK4	Calcium/calmodulin-dependent protein kinase IV
DAMPs	Damage-associated molecular patterns
TRP	Transient receptor potential
HMGB1	High Mobility Group Box 1
